# Plasma Concentrations Predict Aortic Expression of Growth-Arrest-Specific Protein 6 in Patients Undergoing Coronary Artery Bypass Grafting

**DOI:** 10.1371/journal.pone.0079452

**Published:** 2013-11-13

**Authors:** Chien-Hsing Lee, Yi-Shing Shieh, Chien-Sung Tsai, Yi-Jen Hung, Yi-Ting Tsai, Chih-Yuan Lin

**Affiliations:** 1 Division of Endocrinology and Metabolism, Department of Internal Medicine, Tri-Service General Hospital, National Defense Medical Center, Taipei, Taiwan; 2 Department of Oral Diagnosis and Pathology, Tri-Service General Hospital, National Defense Medical Center, Taipei, Taiwan; 3 Division of Cardiovascular Surgery, Department of Surgery, Tri-Service General Hospital, National Defense Medical Center, Taipei, Taiwan; Center for Cancer Research, National Cancer Institute, United States of America

## Abstract

**Aims:**

The tyrosine kinase receptor Axl is expressed in the vasculature, and growth arrest-specific protein 6 (Gas6) is its ligand. Plasma Gas6 levels have been shown to be associated with endothelial dysfunction markers and cardiovascular events. We set out to determine the plasma Gas6 levels in patients undergoing coronary artery bypass grafting (CABG) and investigate the expression of Gas6 and Axl in the aorta.

**Methods and Results:**

Immunoassays were used to investigate plasma Gas6 levels in CABG patients (n = 19) and control subjects (n = 20). The expression of Gas6 and Axl in the injured aorta were examined by reverse transcription-polymerase chain reactions, real-time reverse transcription-polymerase chain reactions, western blotting, and immunohistochemical staining. Plasma Gas6 levels were significantly lower in CABG patients than in matched control subjects. In CABG patients, plasma Gas6 levels were negatively correlated with fasting glucose, E-selectin, and vascular cell adhesion molecule-1 levels. The levels predicted the operative mortality rate and were positively correlated with plasma soluble Axl (sAxl) levels and Gas6 expression in the aorta. Moreover, Gas6 expression was positively correlated with Axl expression in the aorta.

**Conclusion:**

We concluded that plasma Gas6 is associated with fasting glucose, endothelial dysfunction markers, sAxl values, and vascular Gas6 expression in CABG patients, and it predicts the operative mortality of these patients. These findings suggest that the Gas6/Axl system is crucial in vascular biology.

## Introduction

Growth arrest-specific gene 6 (*Gas6*) was first described as one of the genes that was induced during growth arrest in serum-starved fibroblasts [1], but it was later shown to be widely expressed in various cell types, including vascular smooth muscle cells (VSMC), endothelial cells, and monocytes. Gas6, which belongs to the family of plasma vitamin K-dependent proteins, shares 44% identity with anticoagulant protein S at the amino-acid level [2], [3]. Despite some similarities in structure, Gas6 and protein S have different functional properties. Gas6 exerts its effects by binding the three members of the TYRO3, Axl, and Mer (TAM) family. Protein S is also able to interact with TAM receptors, but *in vitro* studies have shown that it has an apparent lower affinity compared with Gas6 [4]. The Gas6/TAM system regulates an intriguing mix of processes that range from leukocyte sequestration and migration, platelet aggregation, and hematopoiesis to proliferation, apoptosis, and phagocytosis. Moreover, the role of the Gas6/TAM system has been perceived to be important in conditions of altered glucose tolerance, injury, inflammation, and repair [5].

Strong evidence, which has been obtained primarily in mouse models, has suggested that the Gas6/Axl signaling pathway is important in the vasculature. Axl is involved in the integrity of the vasculature, and its expression is upregulated at sites of vascular injury, suggesting a role for Axl in vascular remodeling [6]. Gas6 promotes the survival and migration of VSMCs [7], [8], and it has been reported to be overexpressed, along with its receptor Axl, in the rat arterial neointima formation following balloon injury [6]. However, studies of mouse models have led to contradictory results with respect to the pro- or anti-atherogenic properties of Gas6 [9]–[12]. Fewer data in humans are available, and they have been collected mostly in *in vitro* models. Gas6 can exert an anti-apoptotic and chemotactic effect on human VSMCs [8], [13] as well as an anti-inflammatory effect by inhibiting the expression of proinflammatory cytokines in human monocytes/macrophages [14]. Concerning the role of Gas6 in leukocyte adhesion and recruitment, the *in vitro* results that have been obtained in human cells have shown that Gas6 has an anti-inflammatory effect by inhibiting the adhesion of leukocytes to endothelial cells that are stimulated by proinflammatory cytokines [15], which is contrary to the observations that have been made in mice [16]. It has been concluded that Gas6 acts as a protective factor in humans, in part by reducing the proinflammatory phenotype of VSMCs and endothelial cells [17].

Coronary artery bypass grafting (CABG), which is one of the most commonly performed cardiac surgeries, is the preferred revascularization method for the treatment of advanced coronary artery disease that is characterized by persistent inflammation resulting from activated immune cells within coronary lesions and systemic immune responses to the lesions [18], [19]. There is increasing interest in Gas6, given that the Gas6/Axl survival pathway has been implicated in the pathophysiology of atherosclerosis [20]. A previous report by Jiang *et al.* has demonstrated that plasma Gas6 levels correlate with cardiovascular disease, particularly in patients with acute coronary syndrome [21]. Our previous study has also shown that plasma Gas6 protein is associated with altered glucose tolerance, inflammation, and endothelial dysfunction markers [22]. Recently, several reports have demonstrated that the Gas6/Axl system may be involved in the pathogenesis of abdominal aortic aneurysms and critical limb ischemia [23]–[25]. The purpose of the present study was to compare plasma Gas6 levels in CABG patients with those in control subjects and to examine the expression of Gas6 and Axl in the aortic tissues of CABG patients to determine the potential role of Gas6 in CABG patients.

## Materials and Methods

Nineteen patients undergoing CABG for documented coronary artery disease and 20 control subjects without cardiovascular disease were recruited for this study. All CABG patients were hemodynamically stable, and the surgeries were performed electively. The exclusion criteria were an acute myocardial infarction within the previous 2 weeks, unstable angina pectoris, New York Heart Association class-III or -IV congestive heart failure or a left ventricular ejection fraction that was less than 25%, active malignancy, and acute or chronic liver or kidney disease. The study protocol was approved by the Institutional Review Board of Tri-Service General Hospital, Taipei, Taiwan (TSGH097-05-203), and written informed consent was obtained from each patient before participation. Punches of the aortic wall for aortic anastomoses were obtained from the CABG patients. The blood sampling and data collection in the CABG group were conducted before the surgery. The variables for all the patients were collected in order to calculate the EuroSCORE II. The variable definitions were in accordance with the definitions listed on the EuroSCORE website (http://www.euroscore.org/). Operative mortality (%) was calculated with the EuroSCORE system.

### Analytical methods of the plasma samples

After 10 h of fasting, blood samples were obtained to determine plasma glucose, uric acid, aspartate aminotransferase (AST), alanine aminotransferase (ALT), blood urea nitrogen (BUN), and creatinine (Cr) levels and lipid profiles. Plasma circulating E-selectin, intercellular adhesion molecule-1 (ICAM-1), vascular cell adhesion molecule-1 (VCAM-1), and Gas6 proteins were subsequently measured. Serum total cholesterol, triglycerides, and low-density lipoprotein cholesterol (LDL-C) were measured using the dry, multilayer analytical slide method in a Fuji Dri-Chem 3000 analyzer (Fuji Photo Film Corporation, Tokyo, Japan). The intra-assay and inter-assay coefficients of variance (CVs) for LDL-C were 0.9% and 2.6%, respectively. The serum levels of high-density lipoprotein cholesterol (HDL-C) were determined with an enzymatic cholesterol assay method after dextran sulfate precipitation. The intra-assay and inter-assay CVs for HDL-C were 1.2% and 1.8%, respectively. The levels of hemoglobin A_1_c (HbA_1_c) were evaluated with the ion-exchange high pressure liquid chromatography method (VARIANT II, Bio-Rad Laboratories, Inc., Hercules, CA, USA). The intra-assay and inter-assay CVs for HbA_1_c were 1.3% and 2.2%, respectively. Plasma glucose levels were determined by the glucose oxidase method with a Beckman Glucose Analyzer II (Beckman Coulter, Inc., Brea, CA, USA). The intra-assay and inter-assay CVs for glucose were 0.7% and 1.4%, respectively. ALT and AST levels were estimated with a Hitachi 912 Autoanalyzer (Hitachi High-Technologies Corporation, Tokyo, Japan). The intra-assay and inter-assay CVs for ALT and AST ranged from 3.6% to 7.5%. Uric acid and creatinine levels were measured with the ARCHITECT® platform (Abbott Laboratories, Abbott Park, IL, USA). The intra-assay and inter-assay CVs for uric acid were <0.5% and <1.6%, respectively. The intra-assay and inter-assay CVs for Cr were ≤4%. The intra-assay and inter-assay CVs for the BUN assays were 5.0% and 2.5%, respectively. E-selectin, ICAM-1, and VCAM-1 levels were measured with a commercial enzyme-linked immunosorbent assay (ELISA; R&D Systems, Inc., Minneapolis, MN, USA). The intra-assay and inter-assay CVs were 4.4% and 6.0%, respectively, for E-selectin; 3.6% and 7.2%, respectively, for ICAM-1; and 5.2% and 8.5%, respectively, for VCAM-1. Gas6 level was measured by sandwich ELISA with a polyclonal mouse antihuman Gas6 antibody (R&D Systems Europe, Lille, France) as a catcher and a biotinylated goat antiserum as a detector (R&D Systems Europe), as described in our previous study [22]. The method has been validated according to the Food and Drug Administration guidelines in a previous study (intra-assay and inter-assay CVs of 6.5% and 8.5%, respectively; mean recovery for 10 patients of 97%; lower limit of quantification, 0.26 ng/mL). Circulating soluble Axl (sAxl) levels were determined with a RayBio human ELISA kit (RayBiotech, Inc., Norcross, GA, USA) that permits intra-assay and inter-assay CVs of <10% and <12%, respectively [26]. In brief, this commercial kit employs an antibody that is specific for human Axl and is coated on a 96-well plate. Plasma or standards (100 µL) were added for 2 h at room temperature. Washes were repeated, and 100 µL of prepared biotinylated antibody was added for 1 h at room temperature with gentle shaking. Detection was performed with horseradish peroxidase-conjugated streptavidin. All the levels of the above biochemical variables were determined in duplicate, and the values of the two samples were averaged.

### Frozen sections and immunohistochemistry

Human aortic tissues were taken from the operation room and placed immediately in −80°C. The snap-frozen tissues were embedded with OCT (Surgipath, #01480; Leica Biosystems Nussloch GmbH, Nussloch, Germany) and equilibrated at −20°C for frozen sectioning at 5 µm. Sections were air dried for 5 min and washed to remove OCT. For the immunohistochemical staining, each section was blocked with blocking solution for 1 h and incubated for 15 min in 3% H_2_O_2_ that was diluted in methanol, with complete washing of the sections between steps. Specimens of the aorta were stained with primary antibodies [rabbit polyclonal to Axl (abcam plc, Cambridge, UK, #ab37861) and goat polyclonal to Gas6 (R&D System, Inc., #AF885) that was diluted in Dako diluent (Dako Denmark A/S, Glostrup, Denmark, #s3022) for 1 h at room temperature], and this was followed by detection with the Dako REAL EnVision system (Dako Denmark A/S, #K5007) and mounting under cover slips. Using a semiquantitative scale, the staining results were classified into positive (≥25%) or negative (<25%) categories according to the percentage of immunostaining-positive cells. The immunostaining results were evaluated by two investigators (YSS and CHL) who did not have prior knowledge of the patient's clinical status.

### RNA extraction, reverse transcription-polymerase chain reaction (RT-PCR), and real-time RT-PCR

Cryogenic conditions were used for mRNA extraction, which was performed according to the protocol that has been described for the commercial TripureR reaction (Roche Applied Science). Total RNA was extracted, purified, and converted to cDNA with the use of an oligo d(T)_12–18_ primer in order to preserve the relative mRNA profile and produce a template that was suitable for PCR. In the PCR step, 50 pmol each of sense and antisense primers were used. The standard PCR amplification conditions consisted of a hot start at 94°C for 5 min, which was followed by 94°C for 30 s, 55°C for 30 s, and 72°C for 1 min for 30 cycles, with a final amplification at 72°C for 10 min. One-step multiplex quantitative real-time RT-PCR (qRT-PCR) was performed with a CellDirect One-Step qRT-PCR kit (Life Technologies Corporation, Grand Island, NY, USA). A master mix of Taqman MGB primers and probes, reverse transcriptase, and polymerase was prepared and mixed with 40 ng of sample RNA or with a series of known quantities of standard RNA in a final volume of 25 µL. The qRT-PCR reaction was performed in an ABI PRISM 7900HT system (Life Technologies Corporation), with the following cycling conditions: reverse transcription for 15 min at 50°C, 2 min at 95°C, 40 cycles of 95°C for 15 s, and 60°C for 60 s. Each measurement was taken in duplicate, and the threshold cycle value was determined for each amplification curve. The DNA was visualized with ethidium bromide staining, and the band density was determined with a densitometer. The geometric mean of *GAPDH* endogenous expression was used for the normalization of the expression.

### Protein isolation from frozen/OCT-embedded samples

After the excess OCT was washed away with ddH_2_O and 1× phosphate-buffered saline, each tissue section was moved to a 2-mL tube containing ceramic beads (2.8 mm, Bertin Technologies, Montigny-le-Bretonneux, France). Lysis buffer [50 mM Tris-HCl (pH 7.4), 150 mM NaCl, 2 mM ethylenediaminetetraacetic acid, 1% NP-40, 0.1% sodium dodecyl sulfate, and protease inhibitor] was added, and the sample was homogenized by vortexing and grinding in a homogenizer (Precellys®24, Bertin Technologies). The sample was kept on ice for 30 min to complete the lysis reaction and centrifuged (4°C, 13,000× *g*, 15 min) for supernatant collection. The collected sample was stored at −80°C.

### Western blot analysis

Western blotting was performed with a sodium dodecyl sulfate-polyacrylamide gel electrophoresis system. In brief, 20-µg protein samples were resuspended in sample buffer (containing 1 mM dithiothreitol) and electrophoresed on a 10% Tris gel with Tris running buffer. The protein on the gel was blotted onto a polyvinylidene fluoride membrane (PerkinElmer Inc., Waltham, MA, USA). The membrane was sequentially probed with primary antibodies against Axl and Gas6 (both purchased from Santa Cruz Biotechnology, Inc., Santa Cruz, CA, USA), and the β-actin antibody (purchased from Novus Biologicals, LLC, Littleton, CO, USA) was used as a loading control. A horseradish peroxidase-conjugated mouse anti-goat antibody (purchased from Jackson ImmunoResearch Laboratories, Inc., West Grove, PA, USA) was then added as a secondary antibody. Secondary antibodies were detected by autoradiography with enhanced chemiluminescence (ECL, EMD Millipore Corporation, Billerica, MA, USA). Semiquantification of the relative protein expression was performed using Image J (http://rsbweb.nih.gov/ij/).

### Statistical analysis

The descriptive results of the continuous variables are expressed as mean ± standard error of the mean (SE). Before the statistical analysis, the normality of the distribution and the homogeneity of the variables were evaluated by the Levene test for the homogeneity of variance, and the variables were subjected to logarithmic transformation if necessary. We used unpaired *t*-tests for the comparisons of the quantitative variables. The relationships between the variables were tested with a Spearman rank-order correction and partial correlation analysis after adjusting for age. A Bonferroni adjustment for multiple comparisons was analyzed, and the corrected *P* value of 0.025 was considered to represent statistical significance. The statistical analyses were performed using SPSS (version 13.0; IBM Corporation, Armonk, NY, USA).

## Results

### Characteristics of the study population

**Figure 1:**
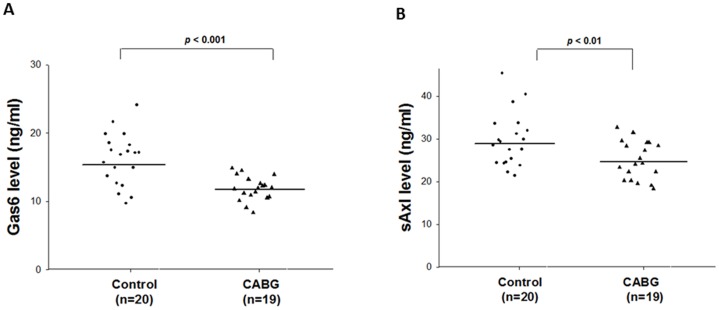
Plasma growth arrest-specific protein 6 (Gas6) and soluble Axl (sAxl) levels in control subjects (Control) and patients undergoing coronary artery bypass grafting (CABG). The lines represent the mean values in each group. The CABG group had significantly lower plasma Gas6 and sAxl levels than the control subject group (*P*<0.0001 and *P*<0.001, respectively).

The main anthropometric and biochemical variables of the 2 groups are summarized in Table 1. CABG patients had significantly higher age and AST, BUN, Cr, total cholesterol, triglycerides, E-selectin, VCAM-1, and ICAM-1 levels than the control subjects. Table 1 and Figure 1 show that plasma Gas6 and sAxl levels were significantly lower (*P*<0.0001 and *P*<0.001, respectively) among CABG patients than among the control subjects.

**Table 1:** Anthropometric and biochemical variables among CABG patients and matched control subjects.

	CABG (n = 19)	Controls (n = 20)	* *P*
Age (years)	66.26±2.68	64.61±2.46	0.782
Sex (M/F)	17/2	17/3	0.842
Diabetes (%)	63	60	0.622
Height (cm)	164.26±1.73	165.82±2.12	0.244
Weight (kg)	72.10±3.12	74.32±3.54	0.782
Blood pressure (mmHg)			
Systolic	135.21±5.28	129.52±4.61	0.221
Diastolic	81.95±1.80	80.19±1.62	0.529
HbA1c (%)	7.62±0.67	7.42±0.71	0.828
Fasting glucose (mmol/L)	8.26±0.22	7.58±0.28	0.214
Uric acid (µmol/L)§	390.19±10.22	375.51±10.47	0.498
AST (U/L)	53.05±10.51	29.11±3.48	**0.005**
ALT (U/L)	27.53±4.63	30.27±5.46	0.572
BUN (mmol/L)	7.33±0.48	6.12±0.29	**0.051**
Creatinine (µmol/L)§	156.47±3.27	85.68±4.71	**<0.001**
Total cholesterol (mmol/L)	5.48±0.47	4.64±0.38	**0.003**
Triglyceride (mmol/L)§	4.80±0.43	3.72±0.32	**0.035**
HDL cholesterol (mmol/L)	1.06±0.04	1.23±0.02	0.098
LDL cholesterol (mmol/L)	3.57±0.19	3.24±0.20	0.302
Endothelial dysfunction markers			
E-selectin (ng/mL)	63.28±2.05	58.48±2.61	**0.025**
VCAM-1 (ng/mL)	589.82±36.48	354.96±46.28	**<0.001**
ICAM-1 (ng/mL)	282.79±21.24	267.52±16.45	**0.017**
Gas6 (ng/mL)§	11.80±0.40	16.11±0.79	**<0.001**
sAxl (ng/mL)	24.89±1.44	29.53±2.28	**0.005**

* assessed by paired *t*-test, data shown as mean ± standard error (*P*<0.05).

§ The logarithms of these variables were used for the analysis.

CABG, coronary artery bypass grafting; HbA1c, Hemoglobin A1c; AST, aspartate aminotransferase; ALT, alanine aminotransferase; BUN, blood urea nitrogen; HDL, high density lipoprotein; LDL, low density lipoprotein; ICAM-1, intercellular adhesion molecule 1; VCAM-1, vascular cell adhesion molecule 1; sAxl, soluble Axl; Boldface indicates statistical significance.

In addition, a correlational analysis was performed in order to evaluate whether other commonly used biochemical markers correlated with Gas6 in the CABG and control groups. In both groups, after adjustment for age, the plasma Gas6 levels were significantly and negatively correlated with fasting glucose, E-selectin, and VCAM-1 levels and positively correlated with sAxl levels (Table 2). In the CABG group, the plasma Gas6 levels were also correlated negatively with the predicted operative mortality rate. The results showed that CABG patients with high plasma Gas6 levels appeared to have lower fasting glucose, endothelial dysfunction markers, and predicted operative mortality rates.

**Table 2:** Age-adjusted Spearman partial correlation coefficients between Gas6 levels and biochemical variables in CABG and control groups.

	CABG (n = 19)*		Controls (n = 20)*	
Variables	r	*P*	r	*P*
Weight (kg)	0.154	0.541	0.218	0.429
HbA1c (%)	−0.305	0.184	−0.521	**0.004**
Fasting glucose (mmol/L)	−0.832	**0.001**	−0.815	**0.001**
Uric acid (umol/L)**^§^**	−0.339	0.183	−0.321	0.176
AST (U/L)	0.081	0.751	0.072	0.823
ALT (U/L)	−0.112	0.659	−0.178	0.592
BUN (mmol/L)	−0.109	0.666	0.121	0.329
Creatinine (umol/L)**^§^**	0.003	0.989	0.012	0.876
Total cholesterol (mmol/L)	−0.060	0.820	−0.089	0.764
Triglyceride (mmol/L)**^§^**	0.262	0.310	0.376	0.084
HDL cholesterol (mmol/L)	0.155	0.714	0.192	0.521
LDL cholesterol (mmol/L)	−0.112	0.791	−0.211	0.433
E-selectin (ng/mL)	−0.685	**0.002**	−0.592	**0.004**
VCAM-1 (ng/mL)	−0.578	**0.012**	−0.602	**0.009**
ICAM-1 (ng/mL)	−0.073	0.774	−0.143	0.394
sAxl (ng/mL)	0.742	**0.002**	0.826	**0.001**
EuroSCORE II	−0.146	0.564	Not applicable	
Operative mortality %	−0.571	**0.017**	Not applicable	

* Corrected for age. **^§^**The logarithms of these variables were used for the analysis. CABG, coronary artery bypass grafting; HbA1c, Hemoglobin A1c; AST, aspartate aminotransferase; ALT, alanine aminotransferase; BUN, blood urea nitrogen; HDL, high density lipoprotein; LDL, low density lipoprotein; ICAM-1, intercellular adhesion molecule 1; VCAM-1, vascular cell adhesion molecule 1; sAxl, soluble Axl; EuroSCORE, European system for cardiac operative risk evaluation. Boldface indicates statistical significance.

### Immunohistochemical staining of Gas6 and Axl of the aorta

**Figure 2:**
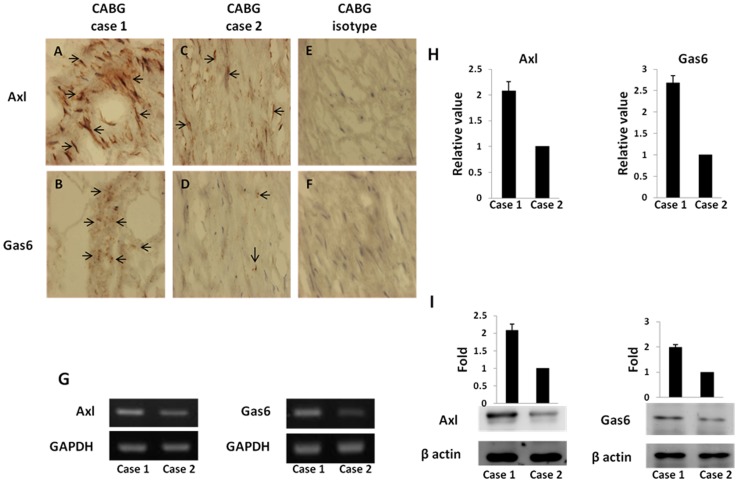
Representative immunostaining, mRNA, real-time polymerase chain reaction, and the expression of Axl and growth arrest-specific protein 6 (Gas6) in cases 1 and 2 in the aorta. Case 1 showed high expression of Axl (A) and Gas6 (B), and case 2 represented low expression of Axl (C) and Gas6 (D). Isotype antibodies for Axl (E) and Gas6 (F) showed negative staining. Similarly, case 1 revealed increased Axl and Gas6 expression compared with that in case 2 in the mRNA (G), real-time polymerase chain reaction (H), and western blotting (I).

In the CABG group, we further examined the distribution and expression of Gas6 and Axl in the aorta with immunohistochemical staining. The results revealed that positive staining for Gas6 and Axl was found predominantly in the tunica intima and tunica media and in the endothelium of the aorta specimens that were analyzed, in which cells showed diffuse cytoplasmic staining for Axl (Figure 2). Using a semiquantitative scale, the staining results of Gas6 and Axl were classified into strong (>25%) or weak (<25%) categories according to the percentage of immunostain-positive cells. The Gas6 and Axl immunoreactivity revealed a positive correlation of the aortic immunostaining of the CABG patients. Thus, strong Gas6 positive staining was accompanied by strong Axl immunoreactivity (Figure 2A, 2B). For weak Gas6 staining, the Axl immunoreactivity was also weak (Figure 2C, 2D).

### Analysis of Gas6 and Axl expression in the aorta by RT-PCR, real-time RT-PCR, and western blot

**Figure 3:**
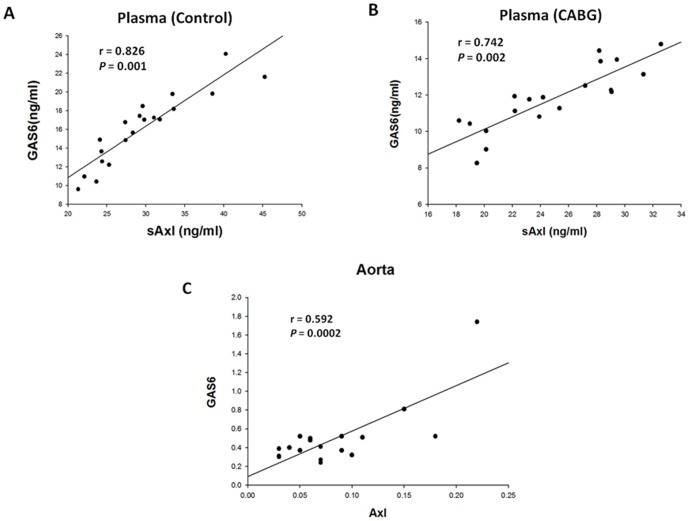
Correlations between plasma sAxl and growth arrest-specific protein 6 (Gas6) levels and between Axl and Gas6 expression in the aorta. The positive correlations between plasma sAxl and Gas6 levels were significant in both groups (A, B). The expression of Gas6 was significantly and positively correlated with Axl protein expression in the aorta (*P* = 0.0002).

To further investigate the role of the Gas6 and Axl, the abundance of Gas6 and Axl in the aorta was determined by RT-PCR, real-time RT-PCR, and western blot analysis (Figure 2G, 2H, and 2I). Similarly, increased Gas6 expression was accompanied by increased Axl expression. For cases with lower Gas6 expression, the Axl expression levels were also lower. These results showed that Gas6 expression was significantly and positively correlated with Axl expression in the aorta (Figure 3C). Moreover, the plasma Gas6 levels revealed a significantly positive correlation with Gas6 expression in the aorta (Table 3). Thus, decreased plasma Gas6 levels suggest inactivation of *Gas6* and Gas6 expression, which was possibly, at least in part, either locally in the aorta or systemically in the vasculature.

**Table 3:** Age-adjusted Spearman partial correlation coefficients between plasma Gas6 levels and tissue expression in CABG group.

	Spearman partial correlation coefficients (n = 19)*	
Variables	r	*P*
Aorta		
Axl/β-actin**^§^**	0.280	0.260
Gas6/β-actin**^§^**	0.512	**0.036**

* Corrected for age. **^§^**The logarithms of these variables were used for the analysis. CABG, coronary artery bypass grafting; Boldface indicates statistical significance.

## Discussion

In this study, we first described that the plasma Gas6 and sAxl levels were significantly lower in CABG patients than in control subjects. In CABG patients, plasma Gas6 levels were negatively correlated with fasting glucose, E-selectin, and VCAM-1 levels. In addition, they predicted the operative mortality rate and were positively correlated with plasma sAxl levels and Gas6 expression in the aorta. Moreover, the immunoreactivity and expression of Gas6 correlated positively with the expression of Axl in the aortic tissue.

Jiang *et al.* have demonstrated that Gas6 levels are significantly lower in stable angina pectoris and acute coronary syndrome groups than in the control group and have indicated a significant correlation between the degree of cardiovascular disease and plasma Gas6 levels [21]. Our previous study has also shown that plasma Gas6 levels are significantly lower among patients with new onset of type-2 diabetes and are associated with glucose levels, inflammation, and endothelial dysfunction markers [22]. Our results demonstrated that the plasma Gas6 levels were significantly lower in advanced coronary artery disease patients undergoing CABG than in control subjects and that plasma Gas6 levels were negatively correlated with fasting glucose, E-selectin, and VCAM-1 levels. These findings reinforced the idea that Gas6/Axl signaling is related to diabetes mellitus and endothelial dysfunction markers, thereby demonstrating that Gas6/Axl signaling may have a crucial role in the pathogenesis of diabetes mellitus and atherosclerosis.

In this study, we found that plasma Gas6 levels were positively correlated with Gas6 expression in the aorta tissues of CABG patients. Our results, which were described for the first time herein, revealed a significant association between systemic circulating Gas6 expression and local tissue Gas6 expression in human advanced vascular disease. Gas6 is derived from endothelial cells, fibroblasts, and vascular smooth muscle cells (VSMCs), and it is aberrantly released into the circulation in response to the disease [7], [17]. The decrease in circulating Gas6 levels may suggest a downregulation of *Gas6* expression, either locally in the aortic tissues or systemically in the vasculature. Interestingly, situations that have been potentially associated with vascular homeostasis either in *in vitro* cell studies or *in vivo* animal models, such as changes in pH, hydrogen peroxide, inorganic phosphate, hypertension, and atherosclerosis, all appear to be affected by Gas6 and/or Axl expression, but the influence is inconsistent [9], [13], [27], [28]. Axl is activated by hydrogen peroxide, which is increased in vascular injury in both VSMCs and *ex vivo* vessels [29]. However, recent studies have demonstrated that inorganic phosphate-induced VSMC apoptosis and subsequent calcification are dependent on the downregulation of the Gas6/Axl/Akt survival pathway that inhibits apoptosis and increases the survival of VSMCs [30], [31]. Furthermore, our unpublished data of the use of an *in vitro* human endothelial cell model elucidated that high glucose levels can lead to downregulation of Gas6/Axl signaling, which in turn causes endothelial dysfunction with a decrease in cell viability and angiogenesis and the induction of monocyte-endothelial cell adhesion by suppressing vascular endothelial growth factor/vascular endothelial growth factor receptor-2 expression and activating adhesion molecules. There is increasing evidence that the persistent inflammation that results from activated immune cells within coronary and aortic lesions and systemic immune responses to the lesions are closely involved in the pathogenesis of advanced coronary artery disease [19]. Recently, several reports have shown that Gas6/Axl signaling intrinsically inhibits inflammatory responses in dendritic cells and macrophages; for example, a TAM triple-knockout mice with low Gas6 levels has shown hyperactivation of monocytes/macrophages [32], [33]. Our results revealed that plasma Gas6 values were lower in CABG patients and positively correlated with Gas6 expression in the aorta tissues. Therefore, we hypothesized that the inflammatory effects of advanced coronary artery disease may be, at least in part, mediated through low Gas6 levels as well as reduced Gas6/Axl signaling, and consequently activated systemic immunity.

Our study revealed that plasma Gas6 levels were not only negatively correlated with the endothelial dysfunction markers E-selectin and VCAM-1 but also negatively correlated with the predicted mortality rate calculated from the EuroSCORE II system. There has been prospective evidence linking endothelial dysfunction with atherosclerosis and demonstrating that endothelial dysfunction was the first step in atherosclerosis [34]. Endothelial dysfunction contributes to cardiovascular diseases, including hypertension, atherosclerosis, and coronary heart disease. EuroSCORE II is a good predictor of operative mortality and morbidity after isolated CABG [35], [36]. Our data first represented that the circulating Gas6 levels were associated with the clinical predicted rate of operative mortality in CABG patients and that it could be used as a nonconventional risk factor in advanced coronary artery disease.

In previous reports, Gas6 and Axl have been widely expressed in many tissues and quite abundantly in the hematopoietic and vascular system [5], [17]. The present study was the first to demonstrate the coexpression of Gas6 and Axl proteins in the aortic tissues of CABG patients by RT-PCR, real time RT-PCR, western blot, and immunohistochemical staining analysis. The biological significance of the coexpression of Gas6 and Axl proteins in the aorta of CABG patients still remains to be clarified, but it may be related to the pathogenesis of advanced cardiovascular disease. Furthermore, our data showed that Gas6 expression was positively correlated with Axl expression in the aorta. It is possible that Gas6 acts on Axl receptor tyrosine kinase signal transduction in aortic tissues in an autocrine fashion.

In contrast to the current study, results from previous studies have shown that circulating Gas6 levels are higher in medical conditions of chronic inflammation, such as chronic renal failure, sepsis, and lupus disease [37]–[39]. One of the possible presumptions is the complexity of the ligand-receptor-downstream pathway process in different physiological or pathological circumstances. For example, the Gas6/TAM system appears to have different stage-specific expression not only in metabolic disorders, such as obesity [26] and type-2 diabetes [22], but also in intravascular thromboembolic processes, such as disseminated intravascular coagulation during sepsis [37], in atherosclerotic plaques of human carotid arteries [40] and in acute coronary syndrome [21]. More longitudinal inspections are required to elucidate the clinical significance of circulating Gas6 levels in the development of metabolic or cardiovascular diseases in human adults. One of the main limitations of the present study was the small size because of the high variability of the Gas6 levels, and further larger studies are required to confirm these results.

## Conclusions

In conclusion, our study indicated that lower plasma Gas6 and sAxl levels were found in CABG patients. In CABG patients, plasma Gas6 levels were associated with glucose levels, endothelial dysfunction markers, and the predicted operative mortality rate. We first revealed that plasma Gas6 levels were positively correlated with the Gas6 expression in the aorta of CABG patients. Gas6, which represents a novel marker in CABG patients, has a critical role in vascular biology. Plasma Gas6 levels may be used as an effective biomarker for atherosclerotic disease.
