# Correction: Plasma Concentrations Predict Aortic Expression of Growth-Arrest-Specific Protein 6 in Patients Undergoing Coronary Artery Bypass Grafting

**DOI:** 10.1371/journal.pone.0175754

**Published:** 2017-04-24

**Authors:** Chien-Hsing Lee, Yi-Shing Shieh, Chien-Sung Tsai, Yi-Jen Hung, Yi-Ting Tsai, Chih-Yuan Lin

Fig 3C is incorrect. In the fourth sentence of the Statistical Analysis section, the authors describe the analyses conducted to produce Fig 3C, “The relationships between the variables were tested with a Spearman rank-order correction and partial correlation analysis after adjusting for age.” However, the r and P value in the Fig 3C are not age-adjusted. Please see the corrected Fig 3 as well as the underlying data for the figure here.

**Fig 3:**
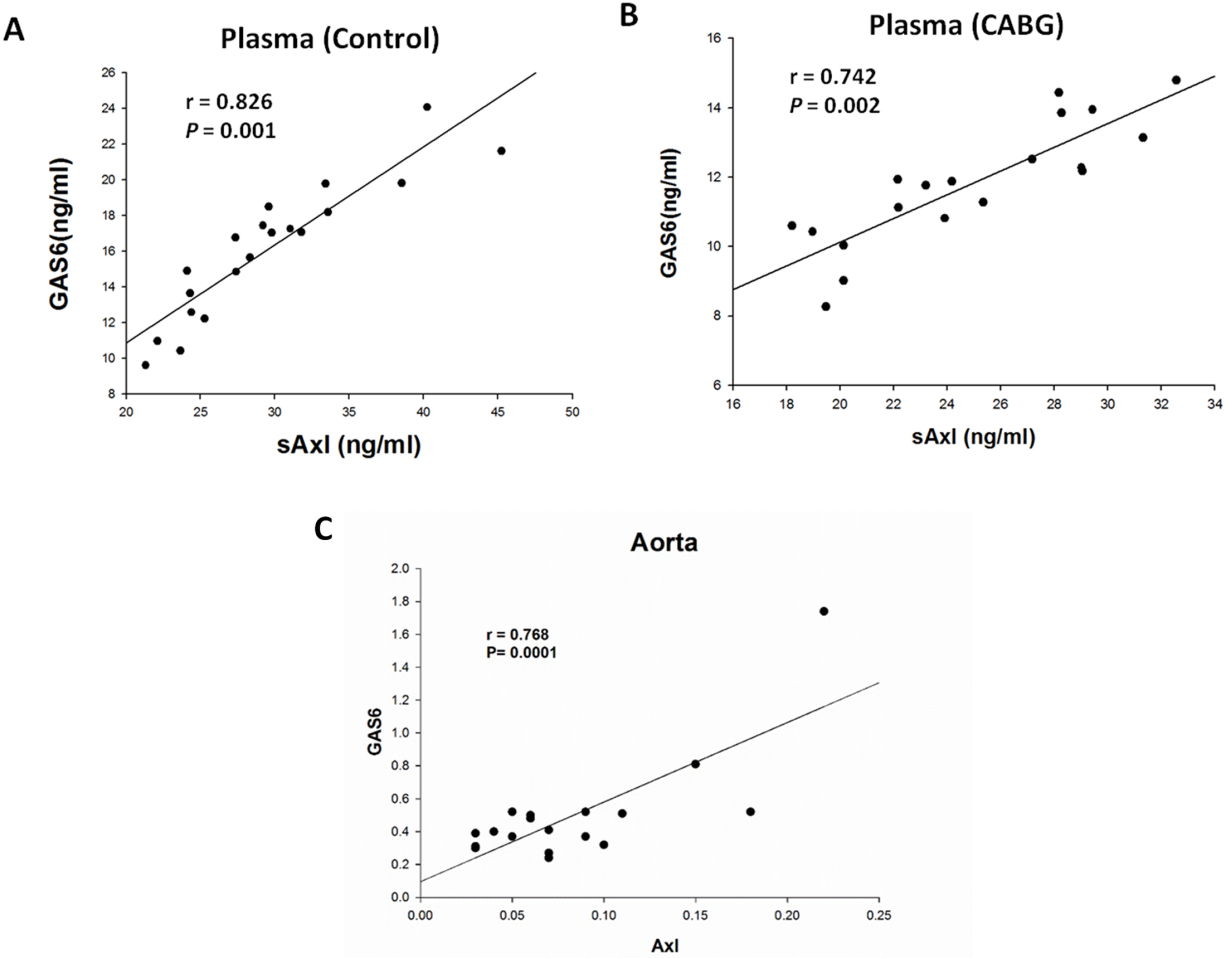
Correlations between plasma sAxl and Growth Arrest-Specific protein 6 (Gas6) levels and between Axl and Gas6 expression in the aorta. The positive correlations between plasma sAxl and Gas6 levels were significant in both groups (A, B). The expression of Gas6 was significantly and positively correlated with Axl protein expression in the aorta (*P*  =  0.0002).

## Supporting information

S1 File Supplementary dataset. This file includes the supplementary data for Fig 3C.
(XLSX)

## References

[1] Lee C-H, Shieh Y-S, Tsai C-S, Hung Y-J, Tsai Y-T, Lin C-Y (2013) Plasma Concentrations Predict Aortic Expression of Growth-Arrest-Specific Protein 6 in Patients Undergoing Coronary Artery Bypass Grafting. PLoS ONE 8(11): e79452 doi: 10.1371/journal.pone.0079452 24236135 10.1371/journal.pone.0079452 PMC3827360

